# Fourier Transform Infrared Spectroscopy (FTIR) and Multivariate Analysis for Identification of Different Vegetable Oils Used in Biodiesel Production

**DOI:** 10.3390/s130404258

**Published:** 2013-03-28

**Authors:** Daniela Mueller, Marco Flôres Ferrão, Luciano Marder, Adilson Ben da Costa, Rosana de Cássia de Souza Schneider

**Affiliations:** 1 Programa de Pós-Graduação em Sistemas e Processos Industriais, Universidade de Santa Cruz do Sul (UNISC), Av. Independência, 2293, CEP 96815-900, Santa Cruz do Sul–RS, Brasil; E-Mails: danielamueller@hotmail.com (D.M.); lucianomarder@unisc.br (L.M.); adilson@unisc.br (A.B.C.); 2 Instituto de Química, Universidade Federal do Rio Grande do Sul (UFRGS), Av. Bento Gonçalves, 9500, CEP 91501-970, Porto Alegre–RS, Brasil; 3 Programa de Pós-Graduação em Tecnologia Ambiental, Universidade de Santa Cruz do Sul (UNISC), Av. Independência, 2293, CEP 96815-900, Santa Cruz do Sul–RS, Brasil; E-Mail: rosana@unisc.br

**Keywords:** HCA, *i*PCA, SIMCA, UATR sensor, biodiesel, raw material, quality control

## Abstract

The main objective of this study was to use infrared spectroscopy to identify vegetable oils used as raw material for biodiesel production and apply multivariate analysis to the data. Six different vegetable oil sources—canola, cotton, corn, palm, sunflower and soybeans—were used to produce biodiesel batches. The spectra were acquired by Fourier transform infrared spectroscopy using a universal attenuated total reflectance sensor (FTIR-UATR). For the multivariate analysis principal component analysis (PCA), hierarchical cluster analysis (HCA), interval principal component analysis (*i*PCA) and soft independent modeling of class analogy (SIMCA) were used. The results indicate that is possible to develop a methodology to identify vegetable oils used as raw material in the production of biodiesel by FTIR-UATR applying multivariate analysis. It was also observed that the *i*PCA found the best spectral range for separation of biodiesel batches using FTIR-UATR data, and with this result, the SIMCA method classified 100% of the soybean biodiesel samples.

## 1. Introduction

Brazil has always stood out on the global scene for its advanced know-how in the production of biofuels, and was the second-largest producer of biodiesel in 2010 and the biggest global consumer in 2011 [1]. The first experiments on the use of ethanol in Otto cycle engines date back to the beginning of the 20th century. Although studies on biofuels in Brazil started long ago, it was only in the 21th century that the country put into action a plan to produce biodiesel on a large scale, taking advantage of the experience acquired with the Pro-Alcohol Program. With the intent to broaden the Brazilian energy matrix, in 2004, the Federal Government launched the National Program of Biodiesel Production and Use (PNPB).

Biodiesel is defined by the National Petroleum Agency (ANP), through Government Directive N° 255, of 15 September 2003, as a compound fuel derived from vegetable oils or animal fats, called B100 [2]. It can be used in pressure-ignited internal combustion engines or for other types of energy generation and can partially or totally replace fossil fuels. Therefore, there are wide possibilities to use biodiesel in urban, road and rail transportation, for the generation of energy, in stationary engines, and others.

Brazil enjoys a privileged position compared to other countries, due to its biodiversity and vast territorial area, able to facilitate the cultivation of distinct species in every region. Consequently, the raw materials for the production of biodiesel can be selected in accordance with their availability in each region throughout the country [3]. Among the sources stand out among them are oilseeds, like cotton, peanut, dendê (palm oil), sunflower, castor bean, barbados nut and soybean [4–6]. Besides the privileged location, two other factors drive Brazil's biodiesel production. The first is the amount of arable land available and the second is the abundance of water resources. According to the Ministry of Agriculture, just considering the new areas that could be destined for the production of oilseeds, they would amount to approximately 200 million hectares [5].

Currently, soybean oil is the most used vegetable raw material for making biodiesel in Brazil, with an average share of 78% in the production of this fuel, followed by cotton oil, with approximately a 4-percent share. The remainder includes animal fats, and other oily materials [7]. Notwithstanding soybean oil's status as most important raw material, in terms of volume, in the production of biodiesel, the Federal Government has been encouraging the development of other oilseed crops, particularly the ones linked with family farming operations. Furthermore, depending on only one crop as major supplier of raw material of an important national energy autonomy project might turn it unsustainable, as it would promote the economic development only (or mainly) of regions where climate and geological characteristics are favorable, whilst keeping the project at the mercy of economic pressures from one production chain only. Similar problems surfaced in the development of the Pro-Alcohol Program in the 1970s.

In this sense, the Ministry of Agriculture, Livestock and Food Supply (MAPA) has been assisting the farmers with crop management practices, providing them with cultivars for the production of biodiesel. In line with this work, the Brazilian government encourages the production of biodiesel from different oilseeds and technological nuances, inviting the participation of agribusiness and family farming operations [5]. Likewise, federal decrees define the taxation rules, which can vary according to planting region, raw material or production category, with distinct tax rates levied on agribusiness and family farming, where the latter is a priority of the program. Another factor that leads to the cultivation of several oilseed crops is easy access to bank loans and reduced interest rates, besides the obligation of the biodiesel producing companies to acquire 5% of their raw material from family farmers. Besides the incentive for the production of biofuels, aligned with the economic development brought about by the production of the oilseeds, the adoption of a quality control program is essential for the identification of the different vegetable oil sources of these biofuels.

This need becomes even more relevant as there are soaring financial attractions for the production of alternative biofuels from renewable sources, in which a diversity of fuel formulations is (or could be) available in the market. This would also inhibit the use of raw materials and the production of biodiesel without the authorization of the regulating organ.

Nevertheless, few studies with the aim to identify a vegetable oil source utilized in the production of biofuels exist. With the incentives of the federal government, now encouraging the use of new raw materials for the production of biodiesel, it is necessary to identify their source and, to this end, there is a need to resort to methodologies that make it possible to identify a vegetable oil source. With regard to chemistry, vegetable oils of distinct sources present a different fatty acids chemical compositions. They differ with regard to the length of the chain, the degree of saturation or the presence of other chemical functions [8], properties that can all be identified through spectrometric techniques [9–14].

A major reason for characterizing its source is related to inspection, as some countries rely on different policies depending on the raw material. Another reason is related to the specific physical-chemical properties of every different vegetable oil and their relation with correct application. Within this context, besides the development of research towards making it technically and economically viable to use other raw materials for the production of biodiesel, it becomes evident (or consequent) that it is necessary to develop analytical techniques to make it possible to identify the vegetable oil source utilized in the production of biodiesel.

Multivariate analyses have recently made possible modeling of chemical and physical properties of simple and complex systems from spectroscopic data. Recent works using near infrared (NIR) spectroscopy, and multivariate analysis for biodiesels in order to identify which vegetable oils are used in production were investigated. Principal component analysis (PCA), and hierarchical cluster analysis (HCA) were used for unsupervised pattern recognition while soft independent modelling of class analogy (SIMCA), was used for supervised pattern recognition [14]. In another work four different multivariate data analysis techniques are used to solve the classification problem, including regularized discriminant analysis (RDA), partial least squares method/projection on latent structures (PLS-DA), K-nearest neighbors (KNN) technique, and support vector machines (SVMs). Classifying biodiesel by feedstock (base stock) type can be successfully solved with modern machine learning techniques and NIR spectroscopy data [15]. Also two classification methods are compared, namely full-spectrum soft independent modelling of class analogy (SIMCA) and linear discriminant analysis with variables selected by the successive projections algorithm (SPA-LDA) [16].

In the other hand, qualitative and quantitative analysis using spectroscopy in the infrared region expanded from the time when the data generated by a FT-IR spectrophotometer could only be scanned, enabling statistical methods to solve problems of chemical analysis [17–21]. In HCA the spectra data matrix is reduced to one dimension, by matching similar pairs, until all points in a single group are matched. The goal of HCA is to display the data in a two-dimensional space in order to emphasize their natural groupings and patterns. The distance between the points (samples and variables) reflects the similarity of their properties, so the closer the points in the sample space, the more similar they are. Results are presented as dendrograms, which samples or variables are grouped according to similarity. In PCA the n-dimensional data is designed into a low-dimensional space, usually two or three. This is done by calculating the principal components obtained by making linear combinations of original variables. In a principal component analysis, clustering of samples defines the structure of data through graphs of scores and loadings, whose axes are principal components (PCs) in which data are designed [22–24]. The *i*PCA analysis consists of dividing the data set into a number of equidistant intervals. For each interval a PCA is performed, and the results are shown in charts of scores. This method is intended to give an overview of the data and may be useful in the interpretation of signs which are more representative of the spectrum to build a good model for multivariate calibration [25–27]. In SIMCA, there is a training set which is modeled by principal component analysis (PCA). Subsequently, new samples are fitted to the model. Test samples are classified as similar or dissimilar [23,28].

## 2. Experimental Section

### 2.1. Materials and Methods

Were used six different vegetable oil sources: canola, cotton, corn, palm, sunflower and soybean. For the latter two, two samples of each oil from different sources were acquired. A two-letter code was used to identify the samples. The first letter specifies if the oil sample is degummed (O) or biodiesel (B), the second letter specifies which vegetable oil source was utilized (for example, C = Canola) and the code that comes next to letter identification represents the analysis reproduction number. Finally, the small letter (a or b) identifies the origin of the sample. The biodiesel samples were produced from samples of degummed oils. From the cotton oil sample two batches were produced and from the soybean sample (b) three batches of biodiesel were produced. This procedure was adopted with the purpose to guarantee the method reproducibility. The canola and sunflower biodiesel batches were acquired from the biodiesel pilot plant of the University of Santa Cruz do Sul—UNISC, in Rio Grande do Sul, Brazil.

The methylation route was used to produce the biodiesel via transesterification. Sodium methoxide (Rodhia) was used as catalyst, and as reagent, methyl alcohol (Vetec, P.A) at a 1:6 molar rate [29]. The biodiesel samples were characterized through methods standardized by the AOCS *Physical and Chemical Characteristics of Oils, Fats, and Waxes* and European Norm (EN) by the following parameters and respective methods: moisture (AOCS Ca2e-84), acidity rate (AOCS Ca5a-40), total glycerol (EN 14105), free glycerol (AOCS Ca14-5) and methanol (EN 14110).

### 2.2. Acquisition of Spectra in the Medium Infrared

The infrared spectra were acquired on a Perkin Elmer model Spectrum 400 FTIR Spectrometer, based on a Universal Attenuated Total Reflectance sensor (UATR-FTIR). A range from 4,000 to 650 cm^−1^ was scanned, with a resolution of 4 cm^−1^ and 32 scans. The crystal utilized in this technique, contains diamond in its upper layer and a zinc selenide focusing element. The spectra of each sample were acquired with six replicates. Later, they were normalized, in order to eliminate the differences in intensity stemming from concentration variations, reducing external effects in the same order of magnitude, and all of them varying within an intensity range from 0 to 1 [30].

### 2.3. Multivariate Data Analysis

All obtained spectra were treated by multivariate analysis tools, using the Hierarchical Cluster Analysis (HCA) and the Principal Components Analysis (PCA) and the Soft Independent Modeling of Class Analogy (SIMCA), through the computer program Pirouette^®^ 3.11 by Infometrix (Bothell, WA, USA). Interval Principal Component Analysis (iPCA) from the software Matlab^®^ 7.11.0 (The Math Works, Natick, MA, USA) was also employed, using the *iToolbox* package (http://www.models.kvl.dk, Copenhagen, Denmark).

### 2.4. Modeling of Biodiesel Batches in the Medium Infrared

**Figure 1.**
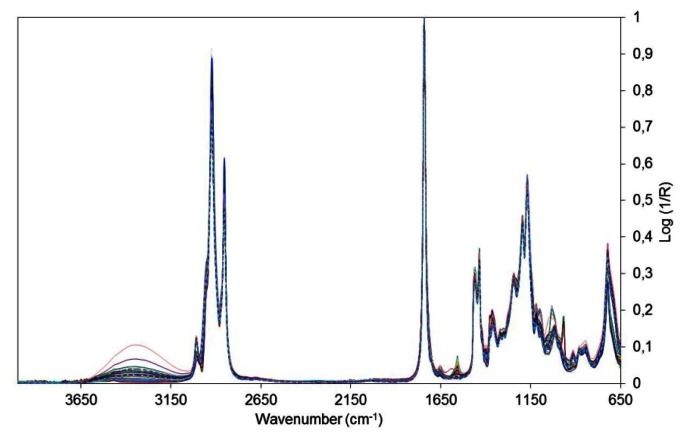
Spectra of samples of biodiesel obtained in the range 4,000 to 650 cm^−1^.

The set of raw spectra of biodiesel samples are shown in Figure 1. To remove noise the spectra were then treated using the Savitzky–Golay first derivative procedure with a second-order polynomial and a 15-point window. Mean centered data and Standard Normal Variate (SNV) were used as pre-processing tools for multivariate analysis [31].

#### 2.4.1. PCA and HCA

**Figure 2.**
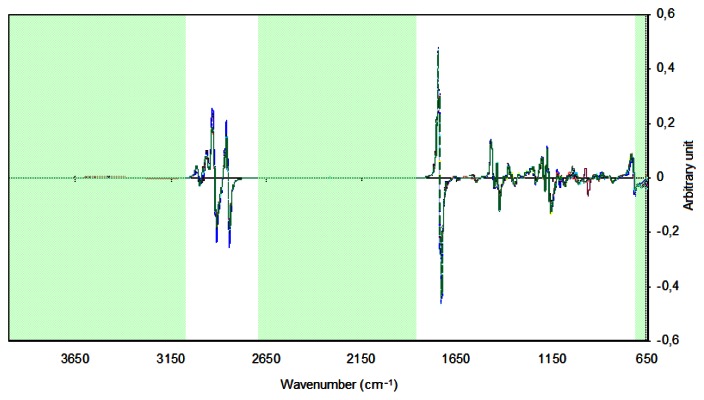
UATR-FTIR spectra of samples of biodiesel with the application of the first derivative and the SNV. The excluded regions of the spectra are highlighted.

In the PCA and HCA, the 735–1,783 and 2,810–3,035 cm^−1^ regions were selected because the other regions contained no spectral information or were polluted by water vapor or carbon dioxide bands due to poor compensation. For obtaining the HCA dendrogram, the Euclidian distance and the incremental connection method were used. In Figure 2, one can observe the spectra of samples of biodiesel with the application of the first derivative and the SNV. The regions of the spectra that were excluded are highlighted.

#### 2.4.2. Interval Principal Component Analysis (iPCA)

The objectives of the results obtained at the Interval Principal Component Analysis (*i*PCA) consisted in detecting the spectral region where there is the best separation of the different samples of biodiesel with the intent to utilize it later in the SIMCA classification method. The spectra were split into 8, 16, 32 and 64 equidistant regions, while the combination of results between the principal components: PC1 *versus* PC2, PC1 *versus* PC3 e PC2 *versus* PC3, was also evaluated.

#### 2.4.3. Soft Independent Modeling of Class Analogy (SIMCA)

Once the best spectral region was obtained with the *i*PCA algorithm, the SIMCA model was built using of the biodiesel spectra data. The SIMCA model built was in accordance with the data in Table 1.

## 3. Results and Discussion

### 3.1. Characterization of the Biodiesel Batches

The results from the characterization of the biodiesel samples are shown on Table 2.

### 3.2. Joint Analysis between the Biodiesel and the Degummed Oil Samples

**Figure 3.**
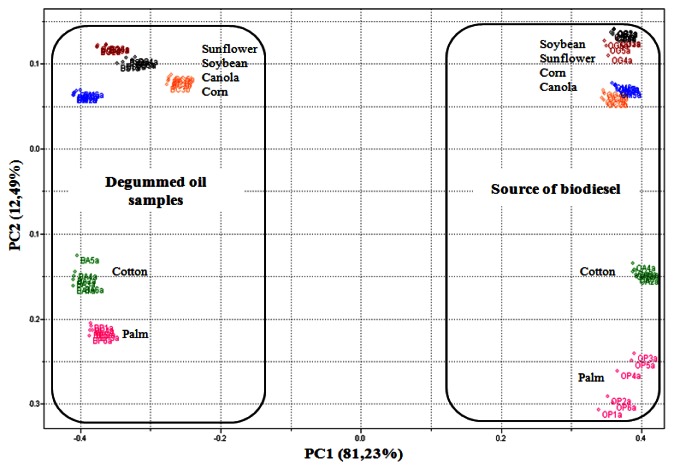
PCA scores plot (PC1 *versus* PC2) for the UATR-FTIR spectra of biodiesel and degummed oil samples.

Through the PCA, it was observed that 93.73% of data variances were explained by the analysis of the two principal components. Figure 3 shows PCA scores plot (PC1 *versus* PC2) obtained from UATR/FTIR data. PC1 separates the biodiesel samples, with positive values, from the degummed oil samples, in negative values on the scores chart. On the other hand, PC2, in turn, manages to separate both the biodiesel samples and the samples of palm and cotton degummed oils, in positive values, from the samples of biodiesel and samples of soybean, sunflower, canola and corn degummed oils**,** in negative values on the scores chart.

**Figure 4.**
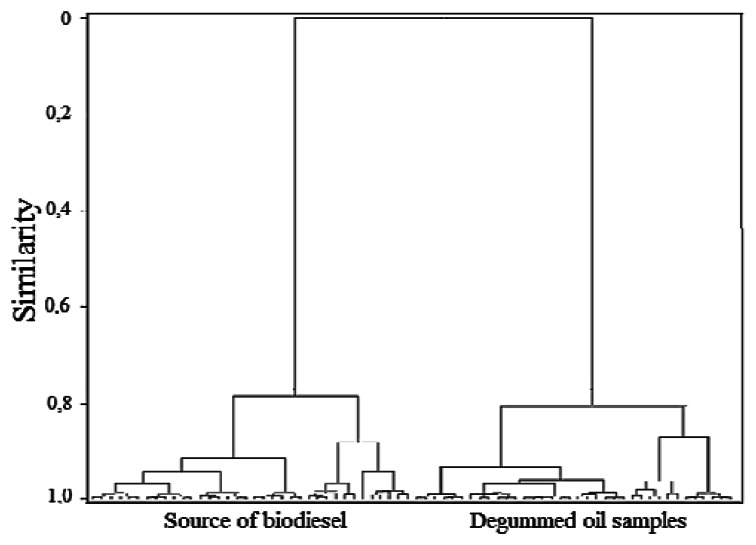
Dendrogram for the UATR-FTIR spectra of biodiesel and degummed oil samples.

Although the samples of degummed oils and the samples of biodiesel are on opposite sides in Figure 3, it is clear that the vegetable oil source exerts an influence on the PC2 of these samples, for example, by observing the samples of biodiesel and the samples of degummed palm and cotton oils, it is ascertained that they are located approximately at the same height of the PC2 axis, though on opposite sides. The same thing also occurs with the other samples. The trends observed through analyses of the principal components were confirmed through the dendrogram obtained by HCA (Figure 4).

In this dendrogram one can observe the presence of two clusters, one associated with the biodiesel samples and the other associated with the degummed oil samples. The results achieved in the dendrogram are totally in line with the results achieved on the PCA scores plot (PC1 *versus* PC2).

### 3.3. Interval Principal Component Analysis (iPCA)

**Figure 5.**
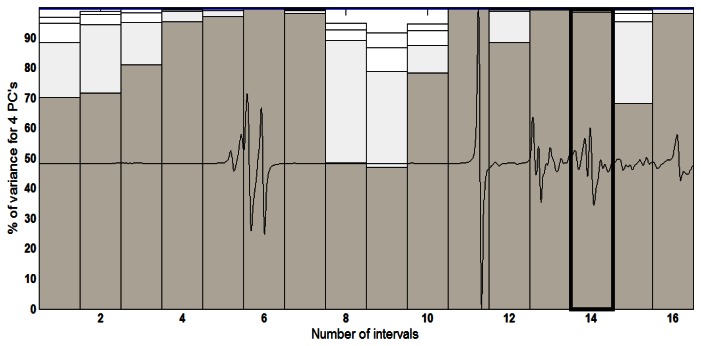
Percent variance to the UATR-FTIR derivate spectra data divided into 16 equidistant intervals.

The best results at the *i*PCA were achieved with the two first principal components (PC1 *versus* PC2) and splitting the spectrum into 16 equidistant intervals. Figure 5 features the percentage variance chart for every region studied. In this chart, for each interval, that is to say, for each region of the spectrum, variance is calculated, in percentage terms, for each principal component. It should be added that the bars present in percentage form (height of the bars) the variance contained in each main component for each interval. In this figure, interval 14 accumulates 99.54% of information in the first two principal components for the UATR-FTIR spectra data.

The spectral region from 1,300–900 cm^−1^ is referred to as the fingerprint, as it confirms the identity of compounds. Within this range, the most important absorptions are the ones stemming from the stretching of the C–O bond of the esters. These absorption ranges of the ester C–O bonds, actually correspond to two asymmetric vibrations that involve the bonds C–C and C–O. In the case of saturated aliphatic esters, the two bands observed appear at 1,275–1,185 cm^−1^ and at 1,160–1,050 cm^−1^. The first involves the bond stretching between the oxygen and the carbonyl carbon, coupled with C–C stretching. The second involves the bond stretching between the oxygen atom and a carbon atom. The band that occurs in the biggest number of waves is usually the more intense of the two [32].

**Figure 6.**
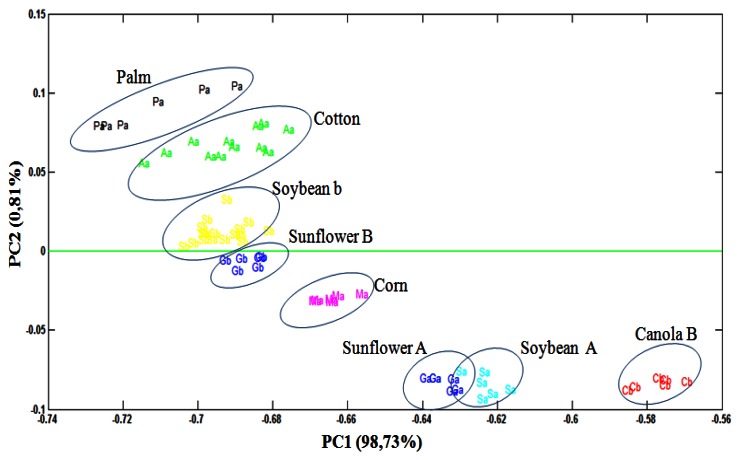
*i*PCA scores plot (PC1 *versus* PC2) for the UATR-FTIR spectra of biodiesel samples.

The spectral region where the best separation of biodiesel samples in the UATR-FTIR spectra data was achieved includes the range of 1,276 to 1,068 cm^−1^, regarding interval 14, which can be visualized in Figure 6.

Figure 6 presents differences between soybean and sunflower samples. It is observed that the batches of soybean A and B are not in the same group and, consequently, they present differences in their chemical composition. This is justified by the characterization data of the biodiesel samples shown on Table 2. The batch of soybean A presents parameters such as moisture, total glycerol, free glycerol and methanol that are not in line with the specified quality patterns of biodiesel (set forth by ANP 07/2008), particularly with regard to the total glycerol rate, reaching the value of 1.72%, and the established limit is 0.25%. The amount of glycerol in the batch suggests that the decantation process was insufficient, which means that the glycerin was not totally removed. In the same way the behavior of the batches of sunflower A and B can be observed, where it becomes evident that the biodiesel from sunflower A has more similarity with the soybean A, which is not in compliance with the recommended specification. For these reasons, the batches of soybean A and sunflower A were not considered in the development of the SIMCA modeling.

### 3.4. Soft Independent Modeling of Class Analogy (SIMCA)

**Figure 7.**
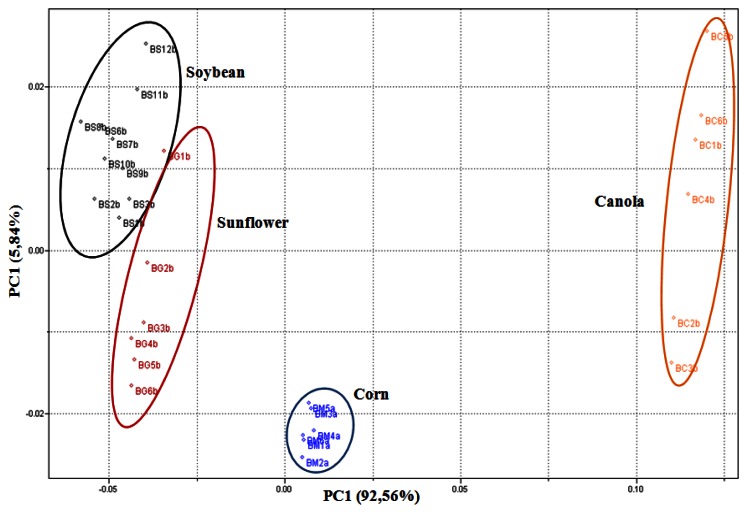
Scores plot (PC1 *versus* PC2) for the UATR-FTIR spectra of biodiesel samples used in the SIMCA training set.

The spectral region, from 1,276 to 1,068 cm^−1^, where the best biodiesel sample separation was achieved using UATR-FTIR spectra data at the *i*PCA was used for the SIMCA modeling. Prior to this modeling, a PCA was developed from the spectra samples that make up the training data presented in Table 1. Upon analyzing the results achieved with the PCA, it was observed that 98.40% of data variance was explained in the two first principal components. The Figure 7 shows the scores plot (PC1 *versus* PC2) for the UATR-FTIR spectra of biodiesel samples used in the SIMCA training set.

**Figure 8.**
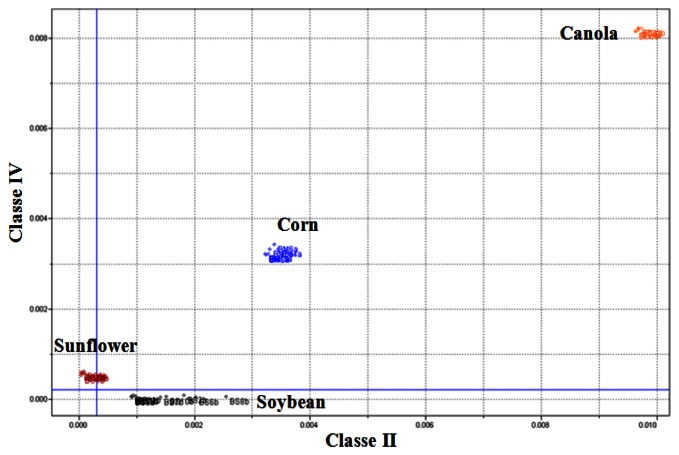
SIMCA Model—Distance between the classes for the training biodiesel samples (Class II *versus* Class IV).

Table 3 presents a summary of the SIMCA model obtained. Figure 8 presents the Coomans diagram which features the orthogonal distances of the biodiesel utilized for the training set. It is observed that Class II and Class IV samples classify correctly into their respective classes.

**Figure 9.**
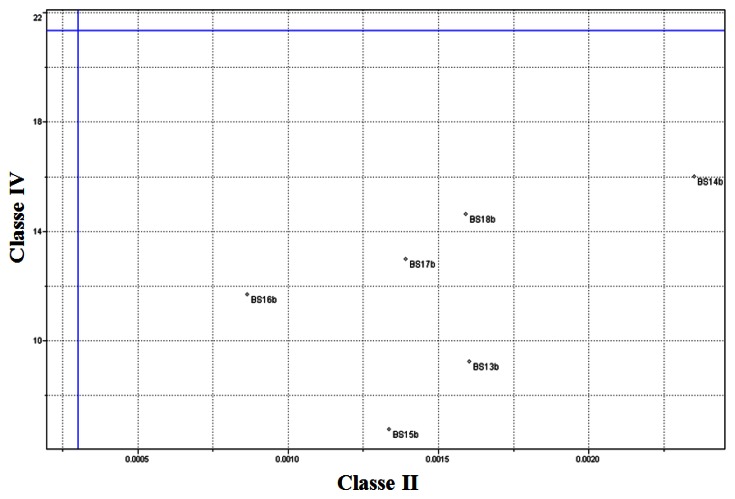
SIMCA Model—Distance between the classes for testing biodiesel samples (Class II *versus* Class IV).

Figure 9 presents the results achieved for the testing samples. The results proved satisfactory and suggest a 100% correct classification for the spectra of the samples of the batch of soybean biodiesel tested.

## 4. Conclusions

The present paper suggests that is possible to develop a methodology to identify vegetable oils used as raw material in the production of biodiesel by Fourier transform infrared spectroscopy using a universal attenuated total reflectance (FTIR-UATR) sensor by applying multivariate methods of analysis. Upon comparing the samples of degummed oils and biodiesel in the FTIR through the PCA, it becomes evident that a vegetable oil source has the same influence on the principal components as the corresponding biodiesel.

The application of principal component analysis by interval method (*i*PCA) made it possible to locate the best spectral intervals for the separation of samples of biodiesel using UATR-FTIR spectra data. In light of the results obtained in the FTIR, the SIMCA modeling allowed for the 100% classification of the soybean biodiesel samples.

## Figures and Tables

**Table 1.** Training and testing data sets of the SIMCA model.

**Training Set**				

**Class**	**Specification Sample**	**Identification**	**Numbers of Batches**	**Number of Spectra**
Class I	Canola	BCa	1	6
Class II	Sunflower	BGa	1	6
Class III	Corn	BMa	1	6
Class IV	Soybean	BSb	2	10

**Testing Set**				

Class IV	Soybean	BSb	1	6

**Table 2.** Results of standardized methods for the characterization of samples of biodiesel.

**Parameters**	**Moisture**	**Index Total Acidity**	**Total Glycerol**	**Free Glycerol**	**Methanol**

**Methods**	**AOCS Ca2e-84**	**AOCS Ca5a-40**	**EN 14105**	**AOCS Ca14-56**	**EN 14110**

**Maximum Standards (ANP 07/2008)**	**500 mg·kg^−1^**	**0.5 mg·KOH·g^−1^**	**0.25% Weight**	**0.02% Weight**	**0.2% Weight**
Soybean A (BSa)	601	0.42	1.72	0.20	0.184
Soybean 1B (BSb)	310	0.35	0.15	0.013	0.154
Soybean 2B (BSb)	326	0.38	0.16	0.009	0.165
Soybean 3B (BSb)	305	0.32	0.14	0.011	0.147
Corn (BMa)	350	0.30	0.22	0.012	0.134
Palm (BPa)	427	0.37	0.23	0.013	0.152
Canola B (BCb)	433	0.51	0.26	0.018	0.179
Cotton A (BAa)	343	0.45	0.15	0.003	0.145
Cotton B (BAb)	356	0.42	0.17	0.006	0.167
Sunflower A (BGa)	458	0.49	0.24	0.009	0.145
Sunflower B (BGb)	489	0.43	0.11	0.012	0.153

**Table 3.** Summary of the results for the SIMCA model.

**Training Data**					

**Class**	**Specification of the Batch**	**Number of Factors**	**% of Cumulative Variance**	**Correctly Classified**	**Incorrectly Classified**
I	Canola	2	66.29	100%	0
II	Sunflower	2	95.79		
III	Corn	2	55.47		
IV	Soybean	3	66.29		
